# Ten simple rules to consider regarding preprint submission

**DOI:** 10.1371/journal.pcbi.1005473

**Published:** 2017-05-04

**Authors:** Philip E. Bourne, Jessica K. Polka, Ronald D. Vale, Robert Kiley

**Affiliations:** 1 Office of the Director, The National Institutes of Health, Bethesda, Maryland, United States of America; 2 Whitehead Institute, Cambridge, Massachusetts, United States of America; 3 Department of Cellular and Molecular Pharmacology and the Howard Hughes Medical Institute, University of California San Francisco, San Francisco, California, United States of America; 4 Wellcome Library, The Wellcome Trust, London, United Kingdom

For the purposes of these rules, a preprint is defined as a complete written description of a body of scientific work that has yet to be published in a journal. Typically, a preprint is a research article, editorial, review, etc. that is ready to be submitted to a journal for peer review or is under review. It could also be a commentary, a report of negative results, a large data set and its description, and more. Finally, it could also be a paper that has been peer reviewed and either is awaiting formal publication by a journal or was rejected, but the authors are willing to make the content public. In short, a preprint is a research output that has not completed a typical publication pipeline but is of value to the community and deserving of being easily discovered and accessed. We also note that the term preprint is an anomaly, since there may not be a print version at all. The rules that follow relate to all these preprint types unless otherwise noted.

In 1991, physics (and later, other disciplines, including mathematics, computer science, and quantitative biology) began a tradition of making preprints available through arXiv [1]. arXiv currently contains well over 1 million preprints. While late to the game [2], the availability of preprints in biomedicine has gained significant community attention recently [3,4] and led to the formation of a scientist-driven effort, ASAPbio [5], to promote their use. As a result of an ASAPbio meeting held in February of 2016, a paper was published [6] that describes the pros and cons of preprints from the perspective of the stakeholders—scientists, publishers, and funders. Here, we formulate the message specifically for scientists in the form of ten simple rules for considering using preprints as a communication mechanism.

## Rule 1: Preprints speed up dissemination

A recent analysis highlighted that the median review time—the time between submission and acceptance of an article—is around 100 days, with a further 25 days or so spent preparing the work for publication [7]. However, these figures—slow as they are—do not include the time researchers spend “shopping around” for a journal to publish their findings, which can induce rounds of editorial rejection before or after peer review. Stephen Royle, a cell biologist at the University of Warwick, undertook an analysis of his published papers over the past dozen years and concluded that the average time from first submission to publication was around 9 months [8]. Royle’s is one example of a well-studied phenomenon [9]. In summary, at a time when technology allows research findings to be shared instantly, the time to access research output appears glacial and similar to the pre-internet era.

## Rule 2: Preprints should be licensed and formatted to facilitate reuse

In principle, preprints can be text and data mined to better comprehend and utilize the knowledge presented. This assumes that copyright, licensing, and format permit such use. Maximizing accessibility and reuse is not necessarily the default currently offered by preprint services. Consequently, when posting a preprint, authors are encouraged to use licenses and formats that facilitate reuse while retaining copyright to their work. Details of copyright, licensing, and format are beyond the scope of this article, but licensing your work as CC-BY (reusable by all, provided attribution is given) and providing a text-accessible version covers most situations. Software tools that facilitate the comprehension of accessible content (for example, Content Mine) are in their infancy but are likely to become mainstream in the next 5–10 years. Better still is the promise that the traditional content of research articles can be integrated with the underlying data, analytics, and commentary to create a new learning experience. To the community, this represents an opportunity to accelerate discovery in ways that are not currently offered by traditional publishers to the contributing authors. Such an offering would presumably provide new opportunities for an author’s work to be used and cited.

## Rule 3: Preprints provide a record of priority

There are a number of resources that provide preprint services to the biosciences (for example, bioRxiv [10], PeerJ Preprints [11], and the Quantitative Biology section within arXiv [12]). All include an uneditable timestamp indicating when the article appeared, which is usually within 24 hours of submission. This date, along with the preprint itself, is made open access (see Rule 2), and thus, anyone (using any internet search engine) can determine the order of priority relative to other published work or, indeed, other preprints. One of the original motivations for creating arXiv was to create a transparent public record of a scientist’s work. By contrast, while journals provide an important service of validation through peer review, establishment of priority can be significantly delayed because the work is not public during the process of peer review in most journals.

The complementary roles of preprints and journals in establishing priority and validation, respectively, are discussed in a commentary by Vale and Hyman [13]. Since preprints may extend beyond traditional published papers, they create an order of priority for these research products as well.

## Rule 4: Preprints do not lead to being scooped

Many scientists wonder if they might be scooped if their work is made public ahead of the formal journal publication. Stepping back, perhaps we should ask: what is the definition of scooping? Here, we take it to mean that, either inadvertently or purposely, an author publishes a biomedical finding and does not provide attribution to the original author(s). The notion that preprints leads to scooping is covered in some detail by ASAPbio [14], and only a synopsis is given here. Again, the presence of arXiv provides a history of what has happened, at least in other disciplines. The short answer, according to Paul Ginsparg, the creator of arXiv, is that intentional scooping is virtually absent in physics because these scientists are aware of the arXiv communication and do not tolerate such behavior. Then, the question becomes whether the biomedical community is somehow different in its ethics or behavior. We believe not, and there is no evidence that this is happening with current preprints. Furthermore, as preprints become more visible and commonplace (like arXiv), scooping will be become increasingly difficult. By contrast, with a nonpublic publication process, it is hard for authors to prove originality during this period if nothing about the work is registered in the public domain. Posters and oral presentations might prove originality, but they are often not publicly and persistently available or detailed enough to support the originality of a body of work. Preprints address this issue, as described in Rule 3, and they can and should be fairly cited.

## Rule 5: Preprints provide access to scholarly content that would otherwise be lost

In addition to our formal publications, as scientists, we have scholarly outputs that we are willing to stand behind but may not have an outlet: a graduate student leaves, gets tied up in a new position, and the paper never gets that final polish yet contains meaningful results and conclusions; a project yields negative data or data that simply does not come together into a coherent story yet has value to the community; replication of a study (or not) represents a useful outcome but is not innovative enough for journal publication. In summary, preprints offer a way of sharing important scholarly output that would otherwise disappear after much time and expense.

Some might argue that work that has not passed peer review should be disregarded. To those, we say, “How much useful information do you get from discussions of unpublished data at meetings, in blogs, and via other forms of non-peer-reviewed content?” We would argue that this type of useful information is growing in both volume and importance. The same naysayers will then likely say, “There is too much misinformation as well as useful information on the internet.” We agree that filters are needed. Human filters will not be able to cope with the volume, hence the need for software tools as described in Rule 2.

## Rule 6: Preprints do not imply low quality

Given that preprints have not been peer reviewed, does that imply low quality? Certainly, the peer review process can add significant value to the work, pointing out errors or areas for improvement. Nevertheless, authors must stand behind their submitted preprint, because it is a public disclosure (and hence a citable entity), albeit a non-peer-reviewed one. Even without peer review, their scientific colleagues will be reading and judging the work, and the authors’ reputations are at stake. Thus, scientists will be careful to disclose their best work that reflects their scientific abilities and expertise, so work of low quality would not be expected. This has been true of arXiv over the years, and the high-quality factor also seems to apply to bioRxiv [10]. To illustrate this, we know a high-profile biomedical research laboratory that now conducts their journal clubs exclusively on preprints [15].

## Rule 7: Preprints support the rapid evaluation of controversial results

Science is, by its nature, iterative and self-correcting. Through preprints, the time to correction can be much reduced. Experience with arXiv has shown that claims concerning, for example, superluminal neutrinos [16] or bicep2 primordial gravitational waves [17] could be discredited before they reached the published literature. In biomedicine, a case in point was the publication of information in May of 2016 [18] that indicated cell phone radiation boosts cancer rates in animals. Given the controversy around such a statement, the National Institutes of Health (NIH) felt an obligation to release all the data, including internal reviews, as quickly as possible so that others could review the findings. This would not be possible through conventional publishing, since neither the form of the manuscript nor the inclusion of an internal review would be suitable for most journals, but a preprint [19] was posted within 24 hours. In a little over 5 months since the preprint was posted, it has been downloaded 148,000 times, providing a more complete picture of the controversial result. It could be argued that the preprint furthered the controversy, but it could also be argued that the authors were under an obligation to provide all available data to describe the research. You could take this further and argue that the science should have been open as it progressed, but that is still not within the comfort zone of most scientists.

## Rule 8: Preprints do not typically preclude publication

Sherpa/Romeo [20] tracks the preprint policies of publishers and their associated academic journals. As can be seen there (and further outlined by [9]), very few journals consider preprints as a “prior form of publication” and reject such manuscripts on the grounds that they had been posted to a preprint server. This is in contrast to the Ingelfinger Rule, enunciated in 1969 by Franz J. Ingelfinger on behalf of the New England Journal of Medicine [21] and followed by many other journals, that would not publish material made available in other media or in other journals. Today, journals publishing papers that have appeared as preprints either speaks to a relaxation of the so-called Ingelfinger Rule or to the idea that preprints are not considered prior publication. In any case, in recent months, more life science journals are developing preprint-friendly policies—and a number have mechanisms to accept journal submissions directly from bioRxiv [16]. We expect this trend to continue as publishers grow to appreciate the value of preprints and how community input can help the author to improve their work and manuscript, leading to a better publication of record.

## Rule 9: Preprints can further inform grant review and academic advancement

The lack of a substantive body of work in support of a particular grant application or academic promotion can be a substantial obstacle to career advancement, particularly for young scientists.

First, consider grant applications to funding bodies. Papers submitted (or even accepted) but not yet published do not help, since the grant reviewer cannot judge the work. By contrast, the availability of preprints can provide a reviewer with the evidence they need to substantiate recent productivity, as well as support the work being proposed in the grant application. It can be argued that this creates more work for the reviewer, but this work results in the ability to perform a more informed review. How individual funders currently treat preprints is variable, and thus, their value to scientists in the way described is also variable. NIH has recently encouraged the inclusion of preprints in grant applications and reports [22]. The Wellcome Trust supports the inclusion of preprints in grant applications and end-of-grant reports [23], the Simons Foundation encourages scientists to post preprints [24], and the Human Frontiers Science Program will allow them to be listed on applications and reports starting in 2017 [25]. Likewise, the Medical Research Council (MRC UK) [26], the Helmsley Charitable Trust [27], and the Canadian Institutes for Health Research [28] are actively encouraging preprints. Currently, many funding agencies are reevaluating their policies (or lack of policies) regarding preprints, so we expect many new pro-preprint policies to emerge in the coming year. Progress of funders in this regard can be tracked from the ASAPbio website [29].

Now consider academic advancement. At the time of academic promotion, a significant body of a scientist’s work could be tied up in the journal review and publication pipeline. Certainly, submitted papers can usually form part of a promotion file, but this carries less weight and credibility than a preprint, which is an acknowledgment by the author that the work is worthy of public viewing and dissemination to the entire scientific community. Moreover, if a knowledgeable reader has significant thoughts on the preprint, those could be posted as commentary, at least on some preprint services. This has wider ramifications, since commentary on preprints may provide the opportunity to improve the final published paper.

## Rule 10: Preprints—one shoe does not fit all

bioRxiv, which is the fastest-growing preprint repository for the life sciences, does not accept preprints that, if posted, could have a damaging effect on human health. This makes sense. Since submissions to bioRxiv only undergo a cursory human review before being posted, there is the possibility that potentially harmful information (e.g., unverified claims about the side effects of vaccines, etc.) or perhaps private and personal information may be revealed. This has ethical, legal, and social issues (ELSI). Such arguments flow into issues of intellectual property (IP) associated with the content of a preprint (noting that IP runs counter to Rule 2), wherein there is the risk of undesirable public release of information. It should be noted that this is not an issue restricted to preprints but one that can apply to talks, posters, etc. too. For research articles, professional editors and reviewers provide additional layers to safeguard from sensitive content being inadvertently released. Currently, preprints have only cursory safeguards, though a future preprint service could enable more rigorous review.

With open content from preprint services available through application program interfaces (APIs), there is the exciting opportunity for researchers to develop tools to better automatically or semi-automatically flag potential ELSI and IP issues. If those tools were open, they would benefit the publishing industry as well.

What should be apparent from these ten simple rules is that the provision and use of preprints in the biomedical sciences is still evolving, but there are clear benefits to the individual and the community. ASAPbio is in the process of developing a governance structure that includes all stakeholders to recommend how best to move forward with the further use of preprints. We invite you to contribute your next paper as a preprint and join the movement.

The original version of this article, prior to peer review, can be found as a preprint here [30].
